# Chiral TiO_2_‑Based Aerogels from Ligand-Imprinted Nanoparticles: Implications for Heterogeneous Asymmetric Photocatalysis

**DOI:** 10.1021/acsanm.6c01351

**Published:** 2026-06-03

**Authors:** Susanna Tinello, Hana Glumac, Markus Niederberger

**Affiliations:** Laboratory for Multifunctional Materials, Department of Materials, 27219 ETH Zurich, Vladimir-Prelog-Weg 5, Zurich 8093, Switzerland

**Keywords:** chiral nanoparticles, TiO_2_, aerogels, self-assembly, chiral imprint

## Abstract

Aerogels composed of chiral nanoparticles combine the high surface area, extensive porosity, and low density of aerogels with the chiroptical and enantioselective properties of nanoscale chiral building blocks, thereby opening up potential applications in photonics, enantioselective catalysis, and molecular recognition. Here, we report the fabrication of a chiral TiO_2_-based aerogel from l/d-threoninol-functionalized nanoparticles. Gelation was induced by controlled destabilization of the nanoparticle dispersion through the addition of a nonsolvent, leading to the formation of a highly porous three-dimensional network. Remarkably, nuclear magnetic resonance (NMR) spectroscopy, supported by Fourier-transform infrared–attenuated total reflectance (FTIR-ATR) spectroscopy, thermogravimetric analysis (TGA), and elemental analysis, reveals that the chiral ligand interacting with the titania surface is largely removed during the gelation and solvent exchange processes. Despite the absence of the ligand, circular dichroism (CD) measurements demonstrate that the nanoparticles constituting the aerogel retain the chiroptical response with a *g*-factor comparable to that of the ligand-functionalized particles. These results indicate that the observed chirality arises from the inorganic TiO_2_ nanoparticles rather than from threoninol, suggesting that chiral structural features were imprinted onto the nanoparticle surface during synthesis. This work demonstrates that chiral information can be preserved within an inorganic aerogel architecture, laying the groundwork for the use of such materials in heterogeneous asymmetric photocatalysis.

## Graphical abstract

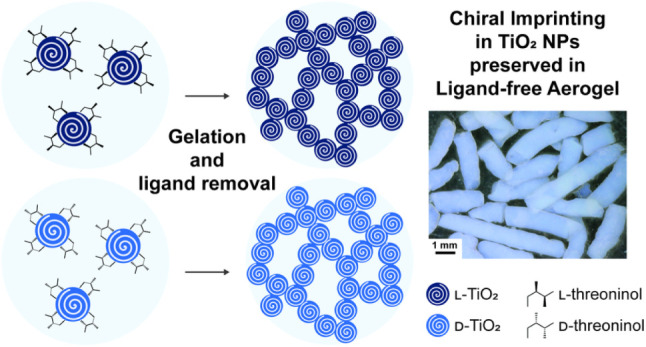

## Introduction

Aerogels are characterized by a finely branched three-dimensional network that provides extremely high porosity and large internal surface areas, making them attractive for applications such as sensing^1^ and catalysis.^2,3^ Traditionally, aerogels have been produced via conventional sol–gel chemistry; however, this approach offers limited flexibility in material design. More recently, an alternative strategy has emerged in which aerogels are assembled from presynthesized nanoparticles.^4,5^ Using this approach, a wide range of materials, including metals,^6–9^ oxides,^10–15^ nitrides,^16^ phosphides,^17,18^ chalcogenides,^19,20^ fluorides,^21^ and their combinations can be processed into aerogels. Because the properties of the nanoscale building blocks are largely preserved during the assembly process, nanoparticle-based aerogels provide exceptional opportunities for tailoring materials to specific applications. In addition to composition, key parameters such as particle size, shape, crystallinity, and surface chemistry can be precisely controlled.^4^ This high degree of design flexibility raises an intriguing question: *can chirality be introduced into nanoparticle-based aerogels?*

Chirality, a fundamental feature in biological systems, is increasingly being integrated into inorganic nanomaterials to exploit their unique optical and catalytic properties. Despite considerable progress in this field, several fundamental questions remain unresolved, particularly regarding the origin of chirality in inorganic systems and its influence on material properties. One particularly intriguing mechanism involves the adsorption of chiral ligands onto inorganic surfaces.^22–24^ In such a system, ligand-surface interaction can distort the surface atomic arrangement, potentially resulting in a chiral structural imprint even after removal of the organic molecules.^22,25^ This concept has significant implications for enantioselective catalysis and chiral separations, as it suggests that inorganic surfaces could retain enantioselective recognition sites independent of the presence of the ligand.

In our previous work,^26^ we demonstrated that chiral titanium dioxide (TiO_2_) nanoparticles can be synthesized through a nonaqueous sol–gel route in the presence of l- or d-threoninol. Notably, after complete removal of the chiral ligand by ultraviolet (UV) light treatment, the TiO_2_ nanoparticles selectively rebound the same enantiomer used during their synthesis from a racemic mixture, exhibiting enantioselective recognition behavior. This result provided strong evidence for the presence of enantioselective active sites on the TiO_2_ surface arising from a structural chiral imprinting effect rather than from the persistence of the chiral organic ligand. Furthermore, these nanoparticles were found to undergo oriented attachment. A similar phenomenon was reported by Niederberger and coworkers^27^ for TiO_2_ nanoparticles synthesized with trizma (2-amino-2-(hydroxymethyl)-1,3-propanediol), an aminoalcohol molecule structurally similar to threoninol. Such anisotropic self-assembly behavior was identified as a key factor in the formation of microporous structures, in which the nanoparticles interconnect to form a three-dimensional network.^15^

Motivated by these findings, we investigate here whether chiral TiO_2_ nanoparticles can be assembled into macroscopic porous architectures while preserving their chiroptical properties. Specifically, we induce gelation of threoninol-functionalized TiO_2_ nanoparticles through controlled destabilization of their colloidal dispersion and subsequently obtain aerogels by supercritical drying. We observed that the chiral ligand is largely removed from the nanoparticle surface during gelation, as demonstrated by nuclear magnetic resonance (NMR) spectroscopy, Fourier-transform infrared–attenuated total reflectance (FTIR-ATR) spectroscopy, thermogravimetric analysis (TGA), and elemental analysis. Remarkably, the resulting nearly ligand-free titania nanoparticles in the aerogel still exhibit a clear chiroptical response when measured by circular dichroism (CD) spectroscopy, with an intensity comparable to that observed for ligand-functionalized nanoparticles. These results strongly indicate that the observed chiral optical activity originates from the inorganic TiO_2_ itself rather than from the presence of the ligand.

In our earlier work,^26^ direct assessment of the chirality of ligand-free TiO_2_ nanoparticles was not possible because UV-induced ligand removal caused irreversible aggregation and poor colloidal stability, preventing reliable CD measurements. Moreover, such UV-treated nanoparticles could not be further processed into gels or aerogels, as gelation requires stable colloidal dispersions. The strategy presented here overcomes these limitations and enables the investigation of chiroptical properties in a fully inorganic aerogel architecture.

In most previously reported chiral gels, chirality originates from the morphology of the assembled structures, typically formed through the use of chiral molecular gelators.^28^ In such systems, chirality is transferred during gelation from the molecules to the supramolecular aggregates through noncovalent interactions. In contrast, the use of intrinsically chiral inorganic nanoparticles as building blocks to generate chiral superstructures has only been explored in a limited number of cases. For example, Nagaoka and coworkers^29^ investigated the assembly of truncated tetrahedral quantum dots, while Zhou et al.^30^ studied the self-assembly of CdTe nanoparticles functionalized with d- and l-cysteine. In their study, the assembly of nanoparticles possessing ligand-induced atomic-scale chirality resulted in the formation of helical supraparticles resembling biological architectures such as the tobacco mosaic virus. Nguyen et al.^31^ incorporated Au@Fe_*x*_O_*y*_ magnetoplasmonic nanowires into titania gels by cogelation, forming a twisted superstructure whose circular dichroism and handedness were controlled by the direction of the magnetic field. Chiral assemblies of plasmonic nanoparticles have also been reported, for instance, in work by Lu et al.,^32^ where gold nanorods were assembled with amyloid fibers for the diagnosis of amyloid-related diseases.

It is important to note that nanoparticles synthesized in solution are rarely purely inorganic, as their surfaces are typically covered by organic ligands.^33,34^ However, in the present system, the organic molecules are mostly removed during gelation and therefore cannot generate chiral supramolecular structures. Instead, the nanoparticles primarily assemble through oriented attachment, as discussed above. For trizma-functionalized TiO_2_ nanoparticles, it has been demonstrated that oriented attachment occurs through the selective replacement of trizma molecules on the {001} facets by water molecules, promoting particle alignment along the [001] direction.^27^ Given the structural similarity between trizma and threoninol, it is reasonable to assume that a similar mechanism governs the assembly of threoninol-functionalized TiO_2_ nanoparticles.

These findings open new perspectives for the development of chiral TiO_2_ aerogels with architectures ideally suited for gas-phase photocatalysis. Importantly, the chirality in these materials originates from the inorganic nanoparticles rather than from organic ligands, which are largely removed during processing. This has two important implications. First, it provides insight into the origin of the chiroptical activity, confirming that it arises from structural features of the TiO_2_ itself. Second, the absence of organic molecules on the surface is advantageous for catalytic applications, as it leaves a larger number of active sites accessible. Consequently, such materials represent a promising platform for the development of enantioselective catalytic systems.

## Experimental Section

### Chemicals and Materials

Titanium tetrachloride (99.9% trace metal basis), anhydrous benzyl alcohol (puriss., >99.0%), ethanol (absolute 99.8% for analysis), ethyl acetate (puriss., >99.7%), and acetone (>99.8% for HPLC) were purchased from Sigma-Aldrich. Heptane (fraction) was purchased from Thommen-Furler AG. l-threoninol (puriss., 97%) and d-threoninol (puriss., 95%) were acquired from Fluorochem. All chemicals were used without further purification.

### Synthesis of l/d **-**Threoninol-Functionalized TiO_2_ Nanoparticles

The nonaqueous sol–gel synthesis of trizma-functionalized titania nanocrystals, originally developed by Niederberger et al.,^27,35^ was subsequently adapted to obtain chiral l- and d-threoninol-functionalized TiO_2_ nanoparticles, following our previously published protocol (Scheme 1).^26^

**Scheme 1:**
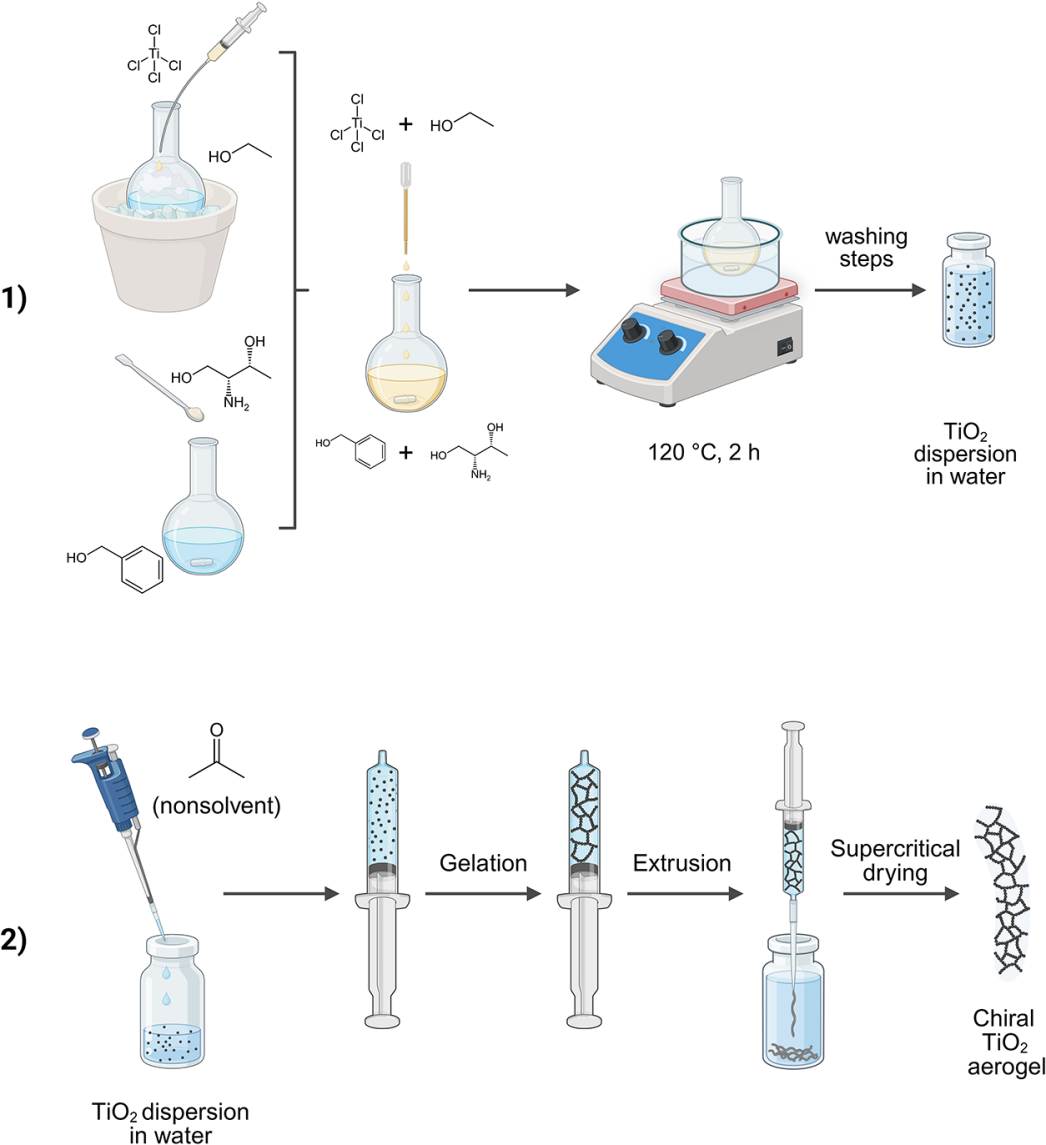
Schematic Illustration of 1) The Synthesis of l/d-Threoninol-Functionalized TiO_2_ Nanoparticles and 2) The Preparation of Chiral TiO_2_ Gels and Aerogels^a^

a Created in BioRender. Tinello, S. (2026) https://BioRender.com/a5lymkx.

In the first step of the synthesis, the ligand was dissolved in benzyl alcohol (40 mL) in a 100 mL round-bottom flask. The ligand mass was adjusted according to the purity of each enantiomer to maintain a titanium-to-threoninol molar ratio of 5:1. The syntheses were performed separately for the l- and d- forms. In the second step of the synthesis, titanium tetrachloride (2 mL, 18 mmol) was added dropwise to ice-cooled ethanol (6.25 mL, 107 mmol) in another 50 mL round-bottom flask under constant stirring at 200 rpm. The addition rate was adjusted so that the released hydrochloric acid fume continuously redissolved in cold ethanol instead of escaping the flask. The resulting yellow ethanolic precursor solution was slowly added to the benzyl alcohol-threoninol mixture under vigorous stirring at room temperature. The mixture was then heated to 120 °C in a preheated oil bath for 2 h under continuous stirring at 500 rpm. During this period, the reaction solution gradually changed from clear yellow to translucent and eventually to milky, indicating the formation of titania nanoparticles. The reaction mixture was finally cooled to room temperature.

For washing, 24 mL aliquots of the reaction solution were mixed with ethyl acetate (7 mL) and heptane (14 mL) to precipitate the particles. The white precipitate was collected by centrifugation for 10 min at 4000 rpm and washed two times with ethyl acetate (35 mL) and subsequently three times with heptane (35 mL). For each washing step, the supernatant was discarded, and the wet precipitate was resuspended in fresh solvent, shaken vigorously, and centrifuged for 3 min at 4000 rpm. After the last washing step, TiO_2_ nanoparticles could either be dispersed in Milli-Q water or dried. To obtain titania nanoparticles dispersed in Milli-Q water, the particles were suspended in heptane (30 mL) before Milli-Q water (7 mL) was added to extract the nanoparticles. The system was stirred gently to maintain the resulting two-phase separation. The functionalized nanoparticles transferred from the heptane phase to the aqueous phase. The aqueous phase containing the TiO_2_ nanoparticles dispersion was extracted with a syringe and stored in a vial. The dried nanoparticles were obtained by vacuum drying at 50 °C for 24 h. They were then ground with an agate mortar and pestle into a fine powder and stored in a vial.

### Preparation of Chiral TiO_2_ Gels and Aerogels

Gelation of the aqueous dispersions was induced by nonsolvent addition, following previously reported protocols (Scheme 1).^2,3,36,37^ Gelation was performed in a granular geometry^3^ by adding 50 vol % acetone to 1 mL of aqueous titania dispersion (110 mg mL^–1^) while vortexing. The resulting destabilized dispersion was transferred into a 5 mL syringe, and the pregelled dispersion was extruded through a nozzle (diameter 1.52 mm) directly into a water-acetone bath containing 70 vol % acetone using a syringe pump at a rate of 2 mL min^–1^, yielding worm-shaped gel granules. The gels were aged in the bath overnight. The next day, the water-acetone bath was replaced every 30 min, increasing the acetone content by 10 vol % at each step until pure acetone (100 vol %) was reached.

After solvent exchange, the pore liquid was removed by supercritical drying with CO_2_ (E3100, Quorum Technologies). The reactor was cooled to 10 °C and filled with liquid CO_2_, then emptied to half capacity and refilled five times. This replacement procedure was repeated three times, with 30 min intervals between cycles, to ensure complete exchange of the pore liquid with liquid CO_2_. After the third cycle, the chamber temperature was raised to 42 °C to reach the supercritical state of CO_2_. Once temperature and pressure stabilized, the system was maintained under supercritical conditions for 30 min at 90 bar. Finally, the pressure was slowly released to atmospheric pressure, and the resulting aerogels were removed and stored under ambient conditions.

## Characterization

### Circular Dichroism Spectroscopy (CD)

CD measurements were performed using a J-815 CD Spectropolarimeter (Jasco). The spectra were measured at 20 °C in quartz cuvettes with a path length of 5 mm. Samples were prepared by dispersing dried nanoparticle powders or ground aerogels in Milli-Q water to a final concentration of 80 μg mL^–1^.

Kuhn’s dissymmetry factor (*g*-factor) allows the conversion of circular dichroism and UV absorbance spectra into a dimensionless *g*-factor spectrum and is defined by the following equation:^38^

$$g=\frac{\Delta A}{A}$$

where Δ*A* = *A_L_* – *A_R_* is the difference between the absorbance of left- and right-handed circularly polarized light, and *A* is the absorbance. Since CD spectrometers report ellipticity (θ) in millidegrees (mdeg), the *g*-factor can also be expressed as

$$g=\frac{\theta }{A\cdot 32980}$$

#### ^1^H Nuclear Magnetic Resonance Spectroscopy (^1^H NMR)

^1^H NMR spectra were acquired on a Bruker 500 UltraShield spectrometer at 500 MHz and processed with MestReNova software. A water suppression method was applied using the watergate W5 pulse sequence with gradients and a double echo to obtain 1D ^1^H NMR spectra. Samples were prepared by dispersing dried nanoparticle powders or ground aerogels to a final concentration of 100 mM in 90 vol% Milli-Q water and 10 vol% D_2_O.

#### Fourier-Transform Infrared Spectroscopy (FTIR)

FTIR spectra were collected using a Bruker Alpha spectrometer equipped with a diamond crystal for Attenuated Total Reflectance (ATR) measurements.

#### Scanning Electron Microscopy (SEM)

SEM images were recorded on a Zeiss GeminiSEM 450 instrument at a voltage of 5 kV. Small aerogel pieces were placed on a sample holder (0.5″ SEM Pin Stubs, 6 mm length, G301F) covered by carbon adhesive discs and sputtered with 3 mm of Pt by a Safematic sputter coater CCU-010.

#### Optical Microscopy

Optical microscopy images were recorded on a Keyence microscope (VHX-6000).

#### Gas Sorption Analysis

Nitrogen gas sorption isotherms were performed at 77 K using a Quantachrome Autosorb iQ instrument. First, the samples were degassed under vacuum at 50 °C for 24 h. Second, the specific surface area of the samples was determined via Multipoint Brunauer–Emmett–Teller (BET) analysis. Density functional theory (DFT) analysis using Non Local DFT calculation model for nitrogen at 77 K on cylindrical pores in silica was used to determine the pore size distribution.

#### Thermogravimetric Analysis (TGA)

Thermal gravimetric analysis and differential scanning calorimetry measurements were performed by using a Mettler Toledo TGA/DSC 3+ Star system. The samples were heated from 20 to 900 °C at a heating rate of 10 K min^–1^ under an air atmosphere.

#### CHN Elemental Analysis

Elemental analysis was performed using Leco Truespec CHN instrument. Before the measurement, the samples were dried under high vacuum at 50 °C for 24 h. The detection limit for each element is 0.2 wt %.

## Results and Discussion

Chiral threoninol-functionalized TiO_2_ nanoparticles were synthesized by a nonaqueous sol–gel method in benzyl alcohol, using metal halides as molecular precursors in the presence of l- and d-threoninol as a chiral ligand, as previously reported.^26^ This procedure yields crystalline anatase TiO_2_ nanoparticles with an average diameter of about 4 nm, exhibiting chiroptical activity in the spectral region where TiO_2_ absorbs. The colloidally stable aqueous dispersion of threoninol-functionalized TiO_2_ nanoparticles served as the starting point for aerogel preparation.

### Gelation of Chiral l/d **-**Threoninol**-**Functionalized TiO_2_

The preparation of nanoparticle-based aerogels relies on the controlled destabilization of the corresponding concentrated colloidal dispersions.^4^ In this study, destabilization was induced by the addition of a nonsolvent, acetone, which lowers the dielectric constant of the aqueous medium. The resulting decrease in dielectric constant reduces interparticle electrostatic repulsion, thereby promoting particle aggregation and gel-network formation. Under these conditions, the nanoparticles first assemble into larger clusters, which subsequently interconnect to form a space-spanning three-dimensional network, ultimately leading to gelation. The resulting gel was then subjected to solvent exchange followed by supercritical drying to obtain the final aerogel. The aerogels were fabricated in a granular form, following the approach reported by Matter et al.^3^ Briefly, the destabilized dispersion was allowed to pregel inside a syringe and then extruded through a nozzle into a solvent bath, forming worm-shaped granules. To ensure rapid reformation of the gel network and prevent redissolution, the nonsolvent fraction in the extrusion bath was increased compared to that in the pregelled dispersion inside the syringe, promoting immediate network stabilization upon extrusion.

Optical microscopy images (Figure 1) show that the worm-shaped granules exhibit a diameter of approximately 1 mm, while their lengths vary between 2 and 8 mm.

**Figure 1:**
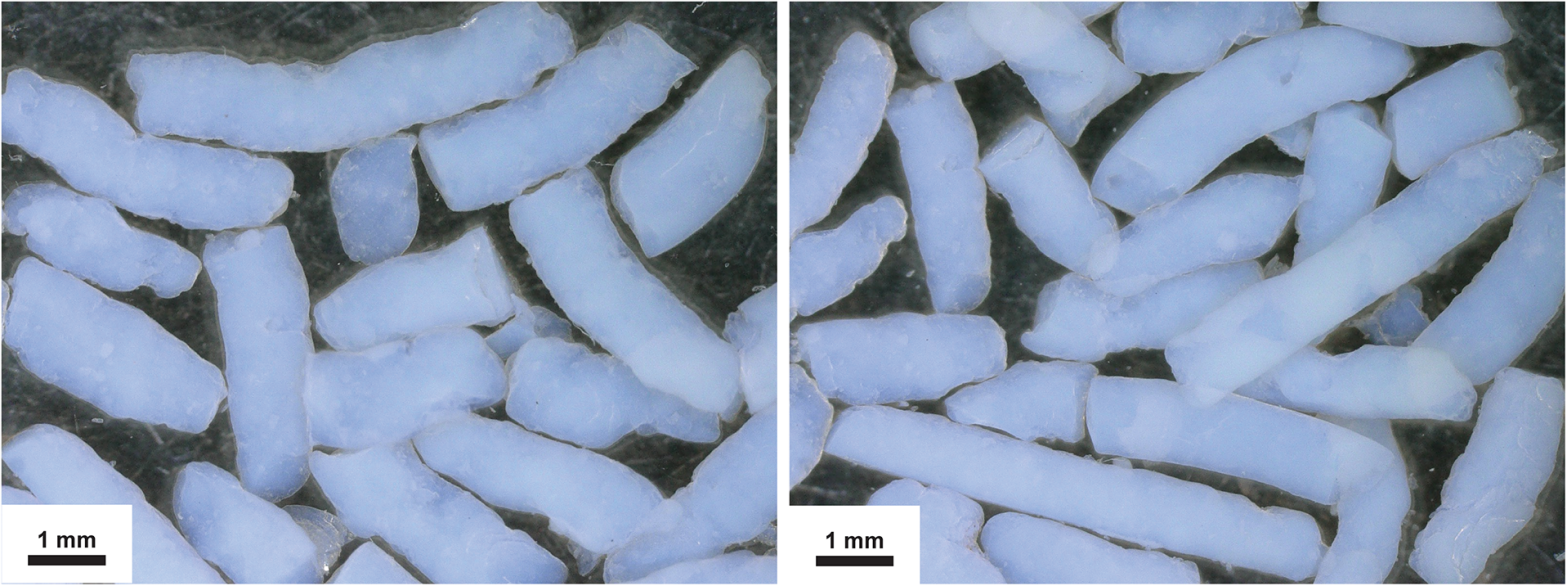
Optical microscopy images of aerogels derived from l-threoninol-functionalized TiO_2_ nanoparticles (left) and d-threoninol-functionalized TiO_2_ nanoparticles (right).

Gas sorption measurements were performed to determine the specific surface area and pore size distribution (Figure S1, Supporting Information). The aerogels display a high specific surface area of approximately 450 m^2^ g^–1^ and a pore size distribution centered around 30 nm, consistent with a mesoporous network formed by interconnected nanoparticles. Scanning electron microscopy (SEM) micrographs reveal a finely branched, open structure (Figure 2 and Figure S2, Supporting Information). In particular, rod-like sections are observed, which can be attributed to the oriented attachment of the threoninol-functionalized TiO_2_ nanoparticles during gel network formation.

**Figure 2:**
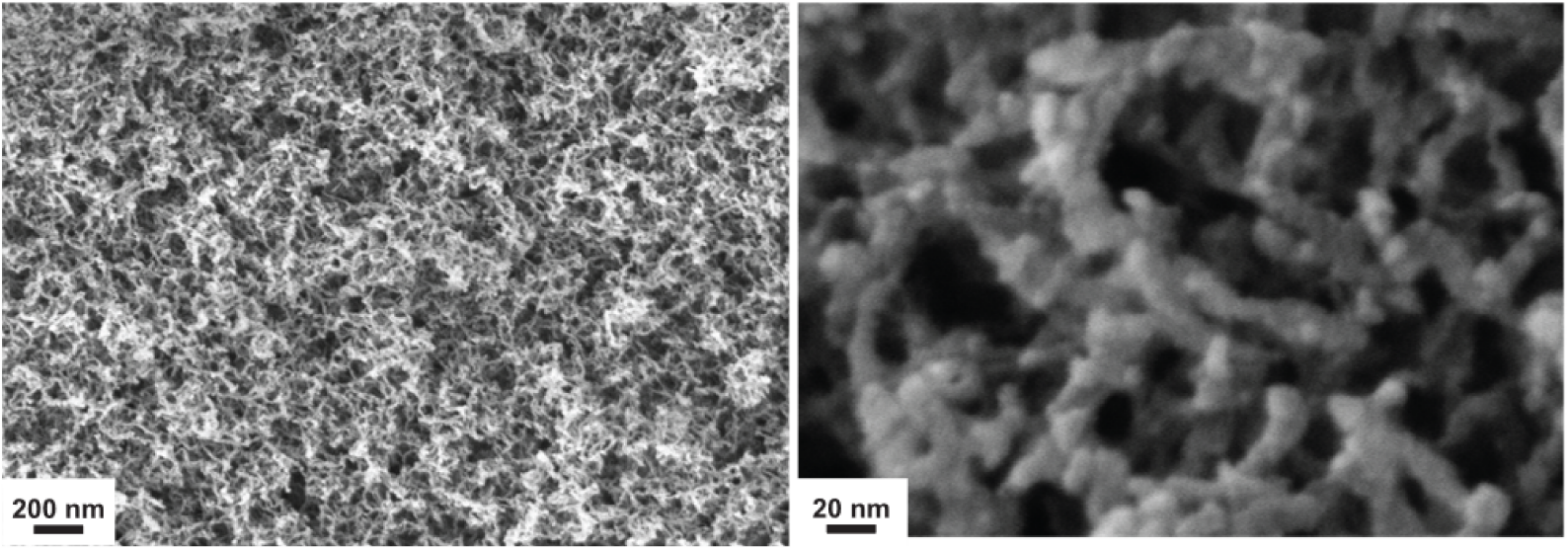
SEM images of aerogels derived from l-threoninol-functionalized TiO_2_ nanoparticles at different magnifications.

### Chiroptical Activity of TiO_2_-Based Aerogels

Although the aerogels preserve size-specific properties of the nanobuilding blocks in the macroscopic material, significant chemical modifications at the surface of the threoninol-functionalized titania may occur during gelation, solvent exchange, and supercritical drying. In particular, the fate of threoninol during the network formation requires careful investigation.

^1^H NMR spectroscopy was performed both on the nanoparticles immediately after synthesis and on the aerogel samples (Figure 3 and Figure S3, Supporting Information). As we described in our previous work, the ^1^H NMR spectrum of the functionalized TiO_2_ nanoparticles displays the characteristic resonances of threoninol. In particular, the following signals are observed in the l-threoninol-functionalized TiO_2_: a doublet at 1.19 ppm (3H, C1), a quintet at 3.84 ppm (1H, C2), a quartet at 3.09 ppm (1H, C3), which appears as a broadened singlet, and two doublets of doublets at 3.62 and 3.76 ppm (2H, C4). All resonances are downfield-shifted and broadened relative to those of the free ligand (Figure S4, Supporting Information). The shift arises from deshielding due to interactions with the TiO_2_ surface, while the broadening reflects reduced mobility and dynamic exchange in the surface-bound state. The broadening may additionally be influenced by the high local acidity at the nanoparticle surface as a result of HCl formation during synthesis. Besides threoninol, the ^1^H NMR spectra reveal the presence of benzyl alcohol (7.32 ppm) and residual ethanol (1.08 and 3.56 ppm), which likely originate from the synthesis procedure. As shown in our previous work,^26^ a broad signal at 7.67 ppm is observed for the threoninol-functionalized system and was assigned to hydroxyl protons of threoninol dynamically interacting with the TiO_2_ surface, most likely through hydrogen bonding to surface sites. Integration of this signal yields an approximate value of 1.5, supporting a dynamic exchange between free and surface-bound hydroxyl groups. Interestingly, the ^1^H NMR spectrum of the corresponding aerogel sample (Figure 3) shows that the ligand signals are no longer present, and only residual solvent peaks are detected. Furthermore, the broad peak at 7.67 ppm is absent in the aerogel spectrum, indicating that hydroxyl-containing organic species are no longer interacting with the TiO_2_ surface.

**Figure 3:**
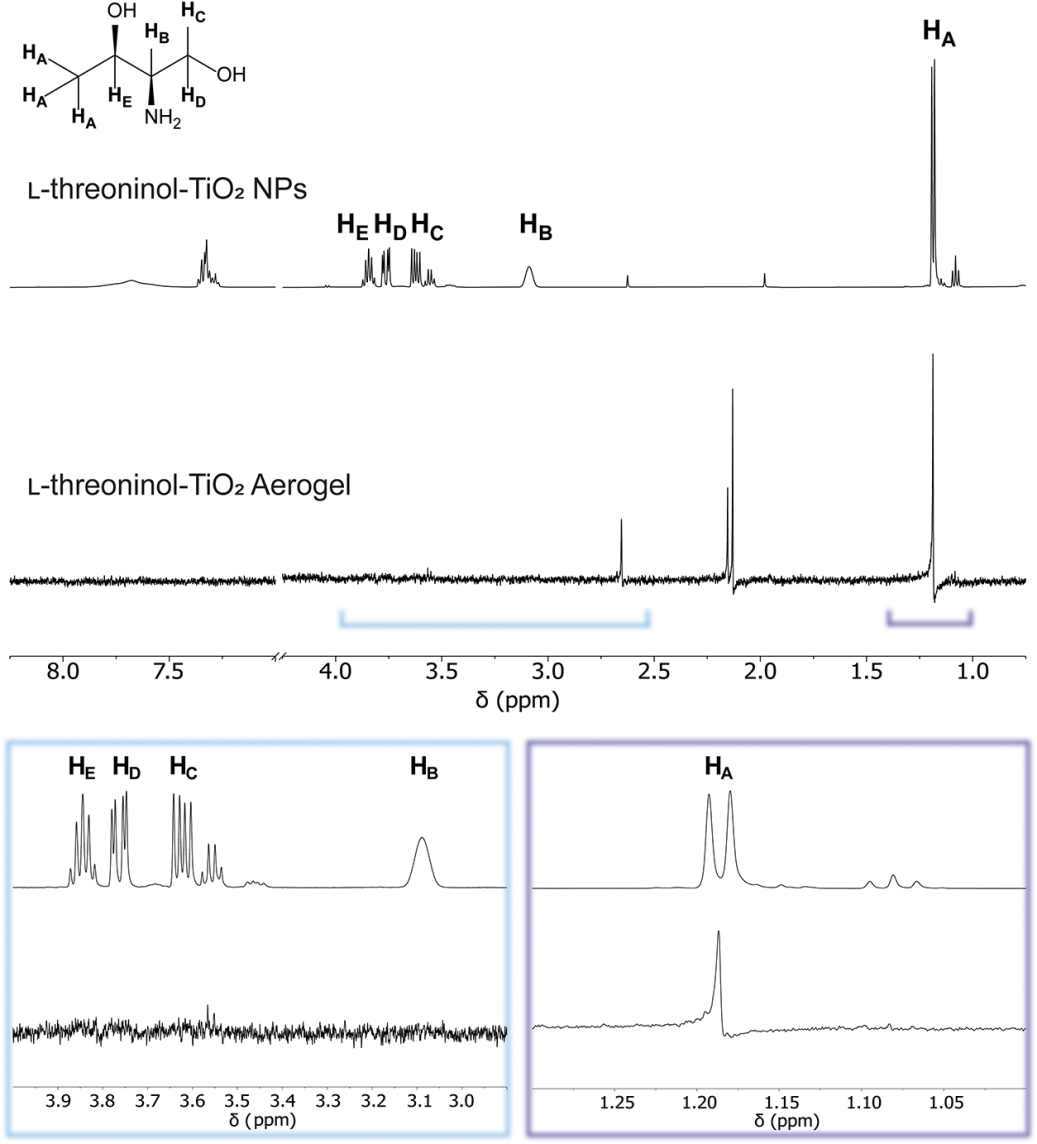
^1^H NMR spectra of l-threoninol-functionalized TiO_2_ nanoparticles (top) and the corresponding aerogel obtained from these nanoparticles (bottom). Zoomed-in regions: 2.9–4.0 ppm (light blue, left) and 1.0–1.3 ppm (violet, right).

These observations strongly suggest that the surface-bound ligand is largely removed during the gelation process, which can be explained by the dynamic interaction of threoninol with the TiO_2_ surface. In our previous work,^26^ we demonstrated the existence of a dynamic chemical exchange between bound and unbound threoninol, supported by diffusion-ordered spectroscopy (DOSY) experiments. The nanoparticle dispersion is primarily stabilized by electrostatic repulsion, as the particles carry a positive charge due to the highly acidic conditions used during synthesis. Threoninol interacts with the titania surface mainly through hydrogen bonding between its hydroxyl groups and surface sites, while the amino group is protonated and positively charged in aqueous dispersion. Since most amino groups are not directly involved in surface binding, they instead contribute to electrostatic stabilization. Owing to the relatively weak and dynamic nature of this interaction, gelation induced by controlled destabilization, achieved by lowering the dielectric constant of the medium, promotes particle–particle interconnection and effectively excludes the ligand from the forming gel network. Specifically, the addition of a solvent with a lower dielectric constant reduces the Debye length, thereby diminishing electrostatic repulsion and allowing attractive van der Waals interactions to dominate.^4^ During subsequent solvent exchange steps, particularly with increasing acetone content, the gel network strengthens, and the loosely bound ligand is progressively removed, dissolving in the acetone bath and later in the liquid CO_2_ during supercritical drying.

Ligand removal is further supported by complementary characterization techniques. Fourier transform infrared (FT-IR) spectra of the aerogel (Figure S5, Supporting Information) show the disappearance of threoninol-related bands, while only residual ethanol signals remain detectable. CHN elemental analysis provides additional confirmation (Table S1, Supporting Information): the nitrogen content, attributed exclusively to the amino group of threoninol, decreases significantly in the aerogel sample from 1.50 and 1.51 wt % to 0.37 and 0.34 wt % for D- and L-threoninol, respectively. It should be noted that the value of 0.35 wt % should be interpreted with caution, as it is close to the detection limit of the instrument (0.2 wt %). Furthermore, short exposure to air during sample preparation could lead to the adsorption of nitrogen molecules onto nanoparticles with such a large surface area. Therefore, despite the significant decrease in nitrogen content, the presence of traces of residual threoninol cannot be completely ruled out. Thermogravimetric analysis (TGA) also supports this finding (Figure S6, Supporting Information). The functionalized nanoparticles exhibit a weight loss of approximately 30%, whereas the aerogel shows a reduced weight loss of about 15%, primarily in the temperature range between 150 and 500 °C. For comparison, pure l-threoninol shows a 90% of mass loss between approximately 150 and 400 °C (Figure S7, Supporting Information). These results indicate a significant decrease in ligand content in the aerogel.

A similar ligand-removal phenomenon during gelation has been reported by Gacoin et al.,^39^ who induced gelation of thiolate-capped CdS nanoparticles using H_2_O_2_. Oxidation of the surface thiolate groups led to their release, exposing reactive sites for nanoparticle condensation and network formation. This gelation approach was then used by Brock et al.^19^ to produce chalcogenide aerogels.

Since the origin of chirality is central to this study, circular dichroism (CD) measurements were performed on the aerogel sample after confirming substantial ligand removal (Figure 4 and Figure S8, Supporting Information). In our previous work,^26^ we demonstrated that TiO_2_ nanoparticles, after ultraviolet (UV) treatment to remove the chiral ligand from their surface, were still capable of selectively rebinding the same enantiomer used during the synthesis from a racemic mixture. However, reliable CD measurements after UV treatment were not possible due to poor colloidal stability and particle precipitation. Moreover, UV-treated particles could not be processed into gels, as gelation requires highly stable dispersions. In contrast, the approach presented here enables reliable CD measurements of nearly ligand-free nanoparticles, because ground aerogel powders form stable colloidal dispersions.

**Figure 4:**
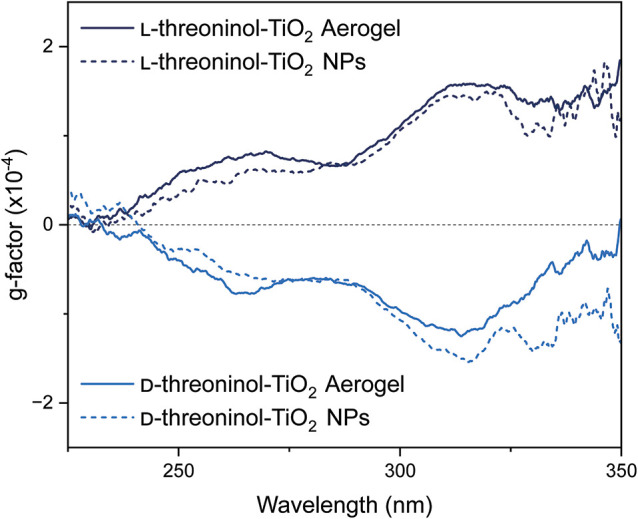
*g*-Factor spectra of l- and d-threoninol-functionalized TiO_2_ nanoparticles (dotted lines) and their corresponding aerogels (solid lines).

Remarkably, the nanoparticles retained their chiroptical activity even after extensive ligand removal. Furthermore, comparison of the *g*-factors of the threoninol-functionalized nanoparticles and the corresponding aerogels shows that the intensity remains essentially unchanged. This is further supported by the quantitative values reported in Table S2, where the *g*-factors at the spectral maxima are listed. If the chiroptical activity depended on the presence of the ligand, a proportional decrease in optical activity with the ligand amount would be expected. In addition, SEM analysis was performed on nanoparticles immediately after synthesis and on those recovered from the ground aerogel (Figure S9, Supporting Information). The images reveal the presence of some aerogel fragments that most likely precipitate in dispersion and do not influence the CD measurement, as well as aggregates of nanoparticles. These aggregates closely resemble those observed in samples of nanoparticles immediately after synthesis in the presence of the ligand. To further exclude morphology- or aggregation-induced chirality, control experiments were performed using aerogels derived from trizma-functionalized TiO_2_ nanoparticles. Trizma is structurally comparable to threoninol, but unlike threoninol, it is achiral. In this case, no chiroptical activity was detected (Figure S10, Supporting Information). Therefore, the observed chiroptical activity in the aerogel cannot be attributed to aggregation effects or morphological changes occurring during gelation and drying.

This observation strongly indicates that the chiroptical activity of the nanoparticles does not result from a ligand-induced effect, in which the highest occupied molecular orbitals (HOMOs) of the chiral ligand would overlap with the valence band (VB) states of TiO_2_.^23,40,41^ Rather, the activity originates from chiral structural features imprinted on the titania surface during nucleation and growth in the presence of the chiral ligand. Thus, while the ligand is essential for establishing this chiral structural imprint during particle formation, its continued presence is not required to maintain the chiroptical response; the structural imprint remains preserved in the inorganic framework even after substantial ligand removal.

## Conclusions

In this study, we report for the first time the fabrication of a TiO_2_-based aerogel that preserves the chiroptical properties of its nanoscale building blocks. Gelation was induced by the addition of a nonsolvent, which promotes interparticle connections and leads to the formation of a three-dimensional network characterized by high porosity and large internal surface area. ^1^H NMR spectroscopy revealed that the chiral ligand interacting with the titania surface is largely removed during the gelation process. This phenomenon enables the direct assessment of the chiroptical activity of the titania nanoparticles that constitute the aerogel framework in the absence of the chiral ligand. Indeed, CD measurements show that the nanoparticles retain their chiroptical activity even after substantial ligand removal, exhibiting a *g*-factor comparable to that observed for ligand-functionalized particles. These results indicate that the observed chirality originates from the inorganic TiO_2_ itself and not from the presence of the organic ligand.

The ability to functionalize nanoparticles with a wide range of chiral ligands and subsequently assemble them into gels offers a versatile approach to the production of aerogels for asymmetric heterogeneous catalysis, avoiding the usual limitation whereby ligands block active surface sites. In this way, the modular assembly of aerogels from preformed nanoparticles introduces an additional design parameter that complements particle size, shape, composition, crystal structure and surface chemistry. This strategy not only expands the toolkit for tailoring material properties, but also enables the integration of photocatalytic activity and stereocontrol within a single catalytic material. An important unresolved question concerns the atomic-level origin of the chiral imprint and its influence on catalytic specificity. In particular, it remains to be determined how closely the structure of a molecule must match the chiral imprint to enable selective recognition and stereocontrol during catalysis. The successful use of chiral imprinting for the separation of enantiomers suggests that these imprinted surfaces can indeed discriminate between closely related chiral structures, providing encouraging evidence that similar effects may be harnessed in catalysis, although the reported separations were performed using the same ligands that were originally employed to generate the chiral imprint. Clarifying this relationship will reveal whether highly tailored imprinted catalysts are required for individual reactions, or whether a single imprinted catalyst can retain stereoselective activity across structurally related substrates.

Overall, this work demonstrates that chiral information can be preserved in a fully inorganic aerogel architecture. These findings open new opportunities for the development of chiral aerogels as heterogeneous catalysts for asymmetric photocatalytic reactions, offering the possibility to combine photocatalysis and stereocontrol within a single catalytic material.

## Supplementary Material



## Notes

Extended material characterization including nitrogen sorption measurements, SEM micrographs, ^1^H NMR spectra, ATR-FTIR spectra, CHN elemental analysis, TGA analysis, and CD and UV–vis spectra (PDF)

## References

[1] Amonette J. E., Matyás J. (2017). Functionalized silica aerogels for gas-phase purification,
sensing, and catalysis: A review. Microporous
Mesoporous Mater..

[2] Kiwic D., Tervoort E., Thach Y. V., Niederberger M. (2026). Mild Synthesis
of Ag-Decorated TiO_2_ Aerogels for Light-Driven CO_2_ Reduction. ACS Mater. Au.

[3] Matter F., Kiwic D., Bernet M., Tervoort E., Niederberger M. (2025). Shaping nanoparticle-based
aerogels for efficient light-driven catalysis. J. Mater. Chem. A.

[4] Matter F., Luna A. L., Niederberger M. (2020). From colloidal dispersions to aerogels:
How to master nanoparticle gelation. Nano Today.

[5] Ziegler C., Wolf A., Liu W., Herrmann A. K., Gaponik N., Eychmüller A. (2017). Modern Inorganic Aerogels. Angew.
Chem., Int. Ed..

[6] Bigall N. C., Herrmann A. K., Vogel M., Rose M., Simon P., Carrillo-Cabrera W., Dorfs D., Kaskel S., Gaponik N., Eychmüller A. (2009). Hydrogels and Aerogels from Noble Metal Nanoparticles. Angew. Chem., Int. Ed..

[7] Herrmann A. K., Formanek P., Borchardt L., Klose M., Giebeler L., Eckert J., Kaskel S., Gaponik N., Eychmüller A. (2014). Multimetallic
Aerogels by Template-Free Self-Assembly of Au, Ag, Pt, and Pd Nanoparticles. Chem. Mater..

[8] Du R., Fan X. L., Jin X. Y., Hübner R., Hu Y., Eychmüller A. (2019). Emerging Noble Metal Aerogels: State
of the Art and a Look Forward. Matter.

[9] Georgi M., Klemmed B., Benad A., Eychmüller A. (2019). A versatile
ethanolic approach to metal aerogels (Pt, Pd, Au, Ag, Cu and Co). Mater. Chem. Front..

[10] Cheng W., Rechberger F., Niederberger M. (2016). Three-Dimensional Assembly of Yttrium
Oxide Nanosheets into Luminescent Aerogel Monoliths with Outstanding
Adsorption Properties. ACS Nano.

[11] Rechberger F., Ilari G., Niederberger M. (2014). Assembly of
antimony doped tin oxide
nanocrystals into conducting macroscopic aerogel monoliths. Chem. Commun..

[12] Rechberger F., Ilari G., Willa C., Tervoort E., Niederberger M. (2017). Processing
of Cr doped SrTiO_3_ nanoparticles into high surface area
aerogels and thin films. Mater. Chem. Front..

[13] Rechberger F., Heiligtag F. J., Süess M. J., Niederberger M. (2014). Assembly of
BaTiO_3_ Nanocrystals into Macroscopic Aerogel Monoliths
with High Surface Area. Angew. Chem., Int. Ed..

[14] Rechberger F., Tervoort E., Niederberger M. (2017). Nonaqueous
sol-gel synthesis of InTaO_4_ nanoparticles and their assembly
into macroscopic aerogels. J. Am. Ceram. Soc..

[15] Heiligtag F. J., Rossell M. D., Süess M. J., Niederberger M. (2011). Template-free
co-assembly of preformed Au and TiO_2_ nanoparticles into
multicomponent 3D aerogels. J. Mater. Chem..

[16] Deshmukh R., Tervoort E., Käch J., Rechberger F., Niederberger M. (2016). Assembly of ultrasmall Cu_3_N nanoparticles
into three-dimensional porous monolithic aerogels. Dalton Trans..

[17] Hitihami-Mudiyanselage A., Senevirathne K., Brock S. L. (2013). Assembly of Phosphide Nanocrystals
into Porous Networks: Formation of InP Gels and Aerogels. ACS Nano.

[18] Hitihami-Mudiyanselage A., Senevirathne K., Brock S. L. (2014). Bottom-Up Assembly of Ni_2_P Nanoparticles into Three-Dimensional Architectures: An Alternative
Mechanism for Phosphide Gelation. Chem. Mater..

[19] Mohanan J. L., Arachchige I. U., Brock S. L. (2005). Porous semiconductor chalcogenide
aerogels. Science.

[20] Arachchige I. U., Brock S. L. (2007). Sol-gel methods
for the assembly of metal chalcogenide
quantum dots. Acc. Chem. Res..

[21] Odziomek M., Chaput F., Lerouge F., Dujardin C., Sitarz M., Karpati S., Parola S. (2018). From Nanoparticle
Assembly to Monolithic
Aerogels of YAG, Rare Earth Fluorides, and Composites. Chem. Mater..

[22] Moloney M. P., Gun’ko Y. K., Kelly J. M. (2007). Chiral highly luminescent
CdS quantum
dots. Chem. Commun..

[23] Tohgha U., Deol K. K., Porter A. G., Bartko S. G., Choi J. K., Leonard B. M., Varga K., Kubelka J., Muller G., Balaz M. (2013). Ligand Induced Circular Dichroism and Circularly Polarized Luminescence
in CdSe Quantum Dots. ACS Nano.

[24] Dolamic I., Knoppe S., Dass A., Bürgi T. (2012). First enantioseparation
and circular dichroism spectra of Au_38_ clusters protected
by achiral ligands. Nat. Commun..

[25] Elliott S. D., Moloney M. P., Gun’ko Y. K. (2008). Chiral Shells and Achiral Cores in
CdS Quantum Dots. Nano Lett..

[26] Tinello S., Emery M., Niederberger M. (2025). Chiral Imprinting
on Inorganic Nanoparticles
for Enantioselective Surface Recognition. Small.

[27] Polleux J., Pinna N., Antonietti M., Hess C., Wild U., Schlögl R., Niederberger M. (2005). Ligand Functionality as a Versatile
Tool to Control the Assembly Behavior of Preformed Titania Nanocrystals. Chem.-Eur. J..

[28] Duan P. F., Cao H., Zhang L., Liu M. H. (2014). Gelation induced supramolecular chirality:
chirality transfer, amplification and application. Soft Matter.

[29] Nagaoka Y., Tan R., Li R. P., Zhu H., Eggert D., Wu Y. M. A., Liu Y. Z., Wang Z. W., Chen O. (2018). Superstructures generated
from truncated tetrahedral quantum dots. Nature.

[30] Zhou Y. L., Marson R. L., van Anders G., Zhu J., Ma G. X., Ercius P., Sun K., Yeom B., Glotzer S. C., Kotov N. A. (2016). Biomimetic Hierarchical Assembly
of Helical Supraparticles
from Chiral Nanoparticles. ACS Nano.

[31] Nguyen H. Q., Nguyen M. C. T., Kang H., Chen H. X., Kiwic D., Matter F., Tervoort E., Niederberger M., Lee J. (2024). Intrinsic Design and Modulated Circular
Dichroism of Inorganic Nanowire/Hydrogel
Composite. Adv. Opt. Mater..

[32] Lu J., Xue Y., Bernardino K., Zhang N. N., Gomes W. R., Ramesar N. S., Liu S. H., Hu Z., Sun T. M., de Moura A. F. (2021). Enhanced optical asymmetry in supramolecular chiroplasmonic assemblies
with long-range order. Science.

[33] Yin Y., Alivisatos A. P. (2005). Colloidal
nanocrystal synthesis and the organic-inorganic
interface. Nature.

[34] Boles M. A., Ling D., Hyeon T., Talapin D. V. (2016). The surface science
of nanocrystals. Nat. Mater..

[35] Polleux J., Pinna N., Antonietti M., Niederberger M. (2004). Ligand-Directed
Assembly of Preformed Titania Nanocrystals into Highly Anisotropic
Nanostructures. Adv. Mater..

[36] Matter F., Niederberger M. (2023). Optimization
of Mass and Light Transport in Nanoparticle-Based
Titania Aerogels. Chem. Mater..

[37] Heiligtag F. J., Leccardi M. J. I. A., Erdem D., Süess M. J., Niederberger M. (2014). Anisotropically
structured magnetic aerogel monoliths. Nanoscale.

[38] Kuhn W. (1930). The physical
significance of optical rotatory power. Trans.
Faraday Soc..

[39] Gacoin T., Lahlil K., Larregaray P., Boilot J. P. (2001). Transformation of
CdS colloids: Sols, gels, and precipitates. J. Phys. Chem. B.

[40] Ben
Moshe A., Markovich G. (2012). Chiral Ligand-Induced Circular Dichroism
in Excitonic Absorption of Colloidal Quantum Dots. Isr. J. Chem..

[41] Govorov A. O., Gun’ko Y. K., Slocik J. M., Gérard V. A., Fan Z. Y., Naik R. R. (2011). Chiral nanoparticle assemblies: circular
dichroism, plasmonic interactions, and exciton effects. J. Mater. Chem..

