# Supplementary material for: Chiral TiO2‑Based Aerogels from Ligand-Imprinted Nanoparticles: Implications for Heterogeneous Asymmetric Photocatalysis
Source: ACS Appl Nano Mater. 2026 Jun 3;9(23):11022–30. doi: 10.1021/acsanm.6c01351 (PMC13270460; doi:10.1021/acsanm.6c01351)
Supplement: Supplementary file 1 [file an6c01351_si_001.pdf]

Supporting Information

Chiral TiO_2-based Aerogels from Ligand-Imprinted Nanoparticles: Implications for Heterogeneous Asymmetric Photocatalysis

Susanna Tinello[a], Hana Glumac[a], Markus Niederberger[a]*

[a] Laboratory for Multifunctional Materials, Department of Materials, ETH Zurich, Vladimir-Prelog-Weg 5, 8093 Zurich, Switzerland

* Email: markus.niederberger@mat.ethz.ch

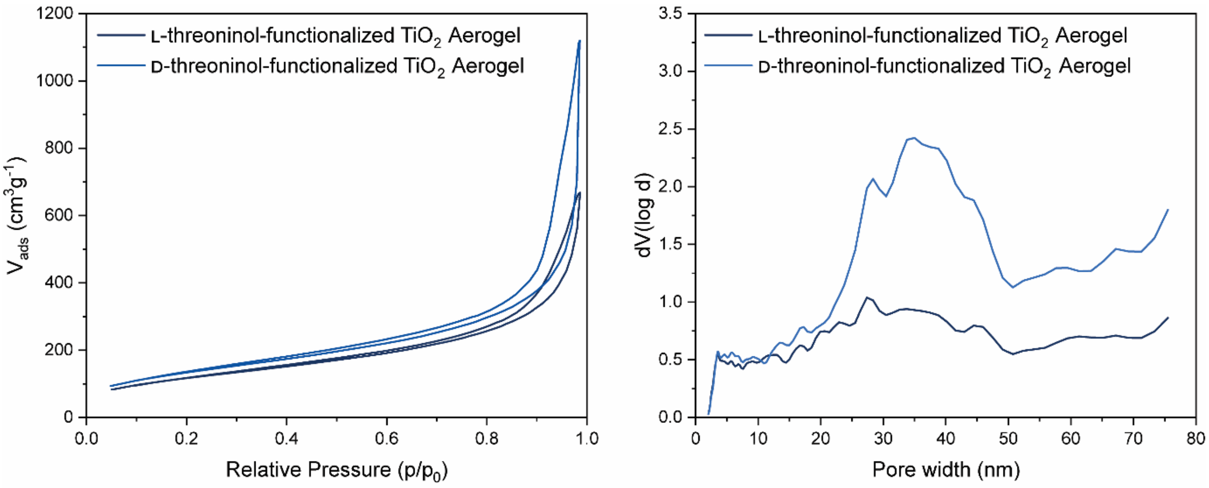

	**Surface area (m^2 g^{-1})**	**Average pore size (nm)**	**Pore volume (cc g^{-1})**
L-threoninol TiO_2 Aerogel	423	27	0.905
D-threoninol TiO_2 Aerogel	485	35	1.425

Figure S1: Nitrogen sorption type IV isotherms (left) and pore size distribution (right) of aerogels derived from L- and D-threoninol-functionalized TiO_2 nanoparticles. The BET surface area, average pore size, and pore volume of the respective aerogels are shown below.

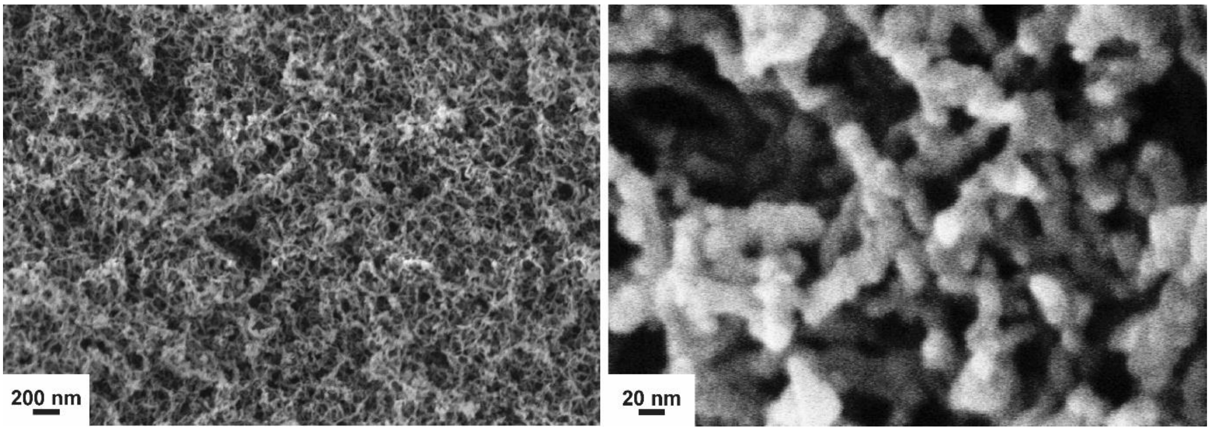

Figure S2: SEM images of aerogels derived from D-threoninol-functionalized TiO_2 nanoparticles at different magnifications.

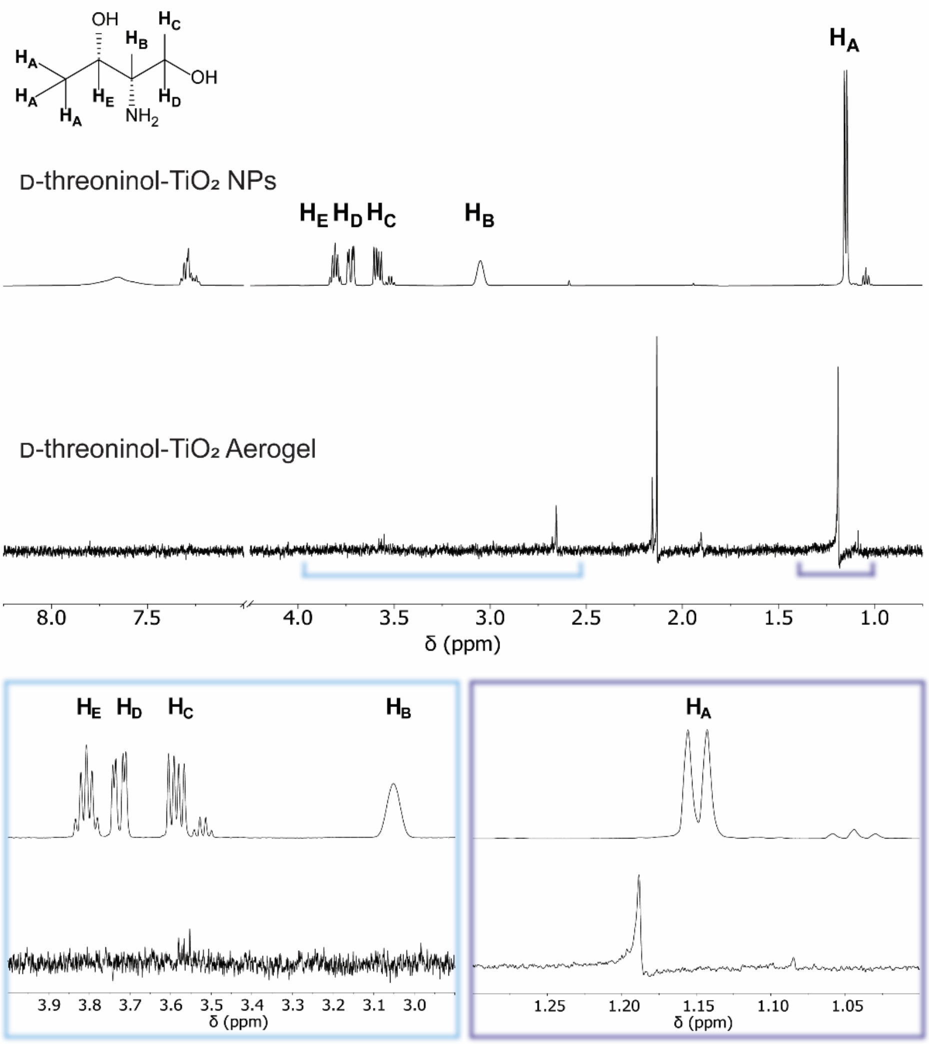

Figure S3: ^{1}H NMR spectra of D-threoninol-functionalized TiO_2 nanoparticles (top) and the corresponding aerogel obtained from these nanoparticles (bottom). Zoomed-in regions: 2.9–4.0 ppm (light blue, left) and 1.0–1.3 ppm (violet, right).

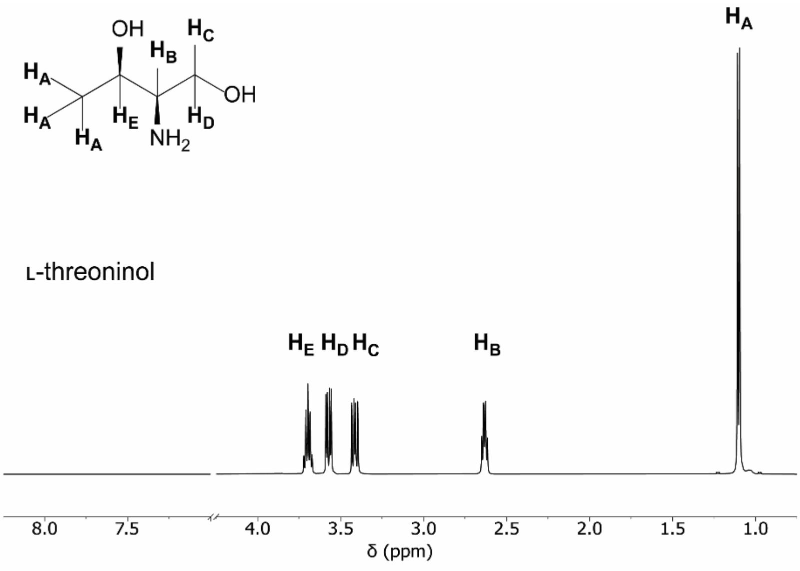

Figure S4: ^{1}H NMR spectrum of L*-threoninol.*

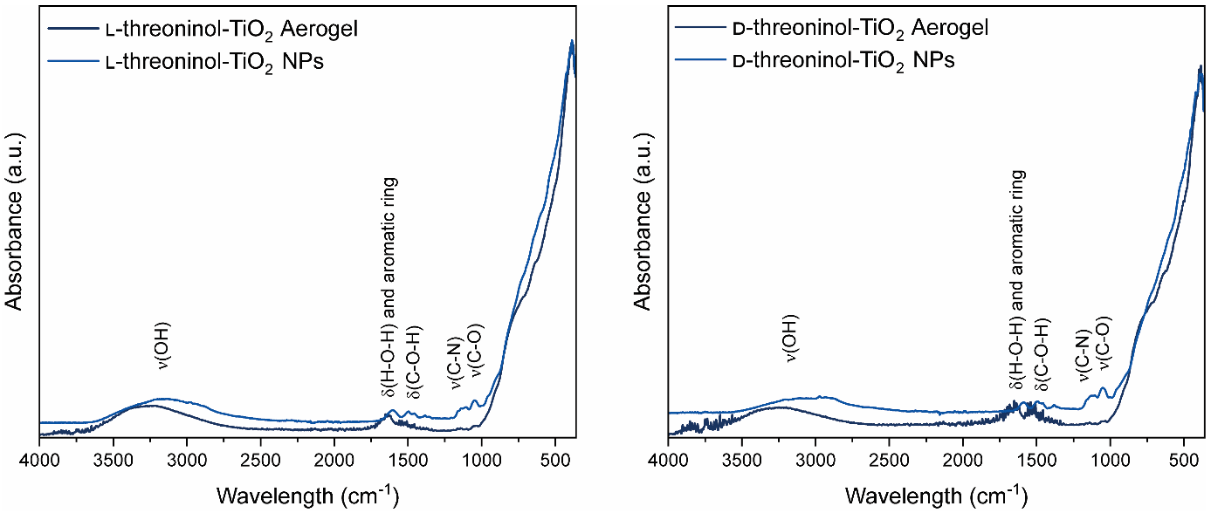

Figure S5: ATR-FTIR spectra of L*-threoninol-functionalized* TiO_2 *nanoparticles and the corresponding aerogel (left), and* D*-threoninol-functionalized* TiO_2 *nanoparticles and the corresponding aerogel (right).*

	C (wt.%)	H (wt.%)	N (wt.%)
L-threoninol TiO_2 NPs	8.03	2.30	1.51
L-threoninol TiO_2 Aerogel	2.50	1.79	0.34
D-threoninol TiO_2 NPs	9.27	2.29	1.50
D-threoninol TiO_2 Aerogel	2.76	1.83	0.37

Table S1: CHN elemental analysis of L- and D-threoninol-functionalized TiO_2 nanoparticles and their corresponding aerogels.

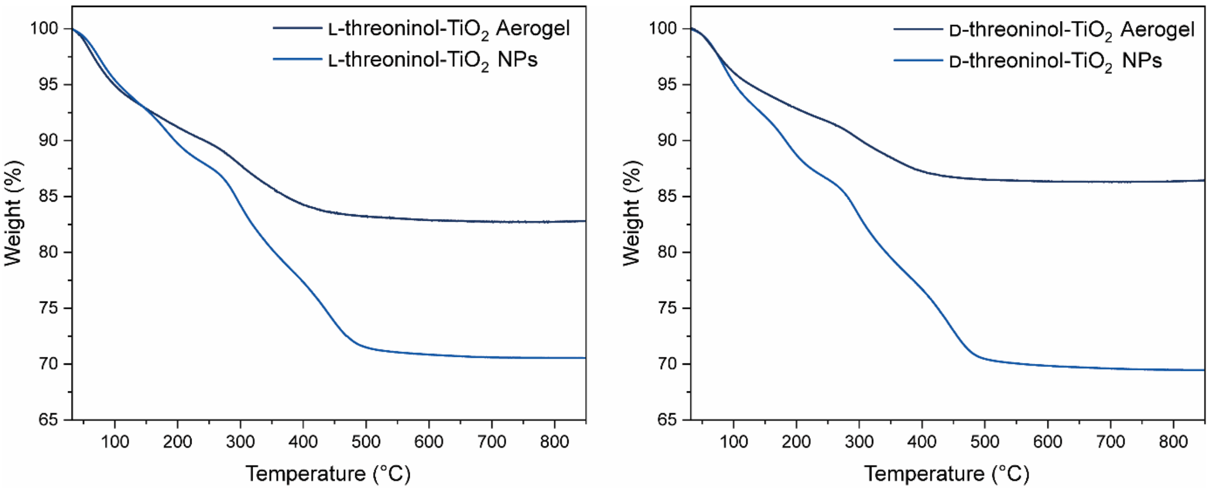

Figure S6: Thermogravimetric analysis (TGA) of L-threoninol-functionalized TiO_2 nanoparticles and the corresponding aerogel (left), and D-threoninol-functionalized TiO_2 nanoparticles and the corresponding aerogel (right).

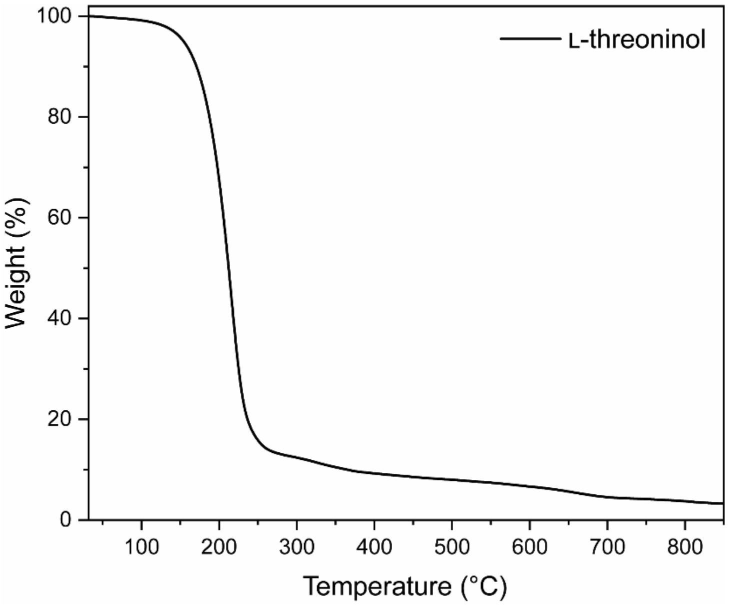

Figure S7: Thermogravimetric analysis (TGA) of L-threoninol.

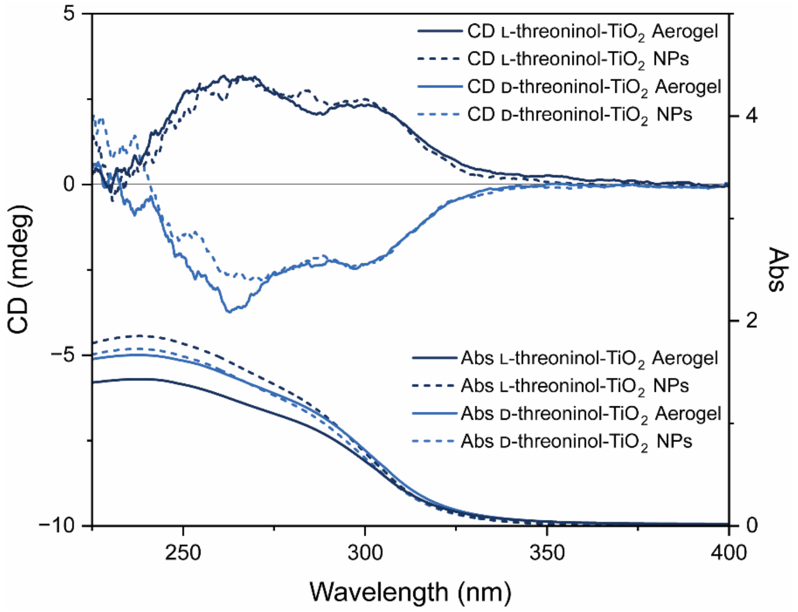

Figure S8: CD spectra and UV–vis spectra of L- and D-threoninol-functionalized TiO_2 nanoparticles (dotted lines) and their corresponding aerogels (solid lines).

	L-threoninol TiO_2 NPs	L-threoninol TiO_2 Aerogel	D-threoninol TiO_2 NPs	D-threoninol TiO_2 Aerogel
g_{265}	$5.9x10^{-5}$	$7.9x10^{-5}$	$-5.5x10^{-5}$	$-7.6x10^{-5}$
g_{310}	$1.4x10^{-4}$	$1.5x10^{-4}$	$-1.4x10^{-4}$	$-1.2x10^{-4}$

Table S2: g-factor intensities at 265 nm and 310 nm for the nanoparticle and aerogel samples.

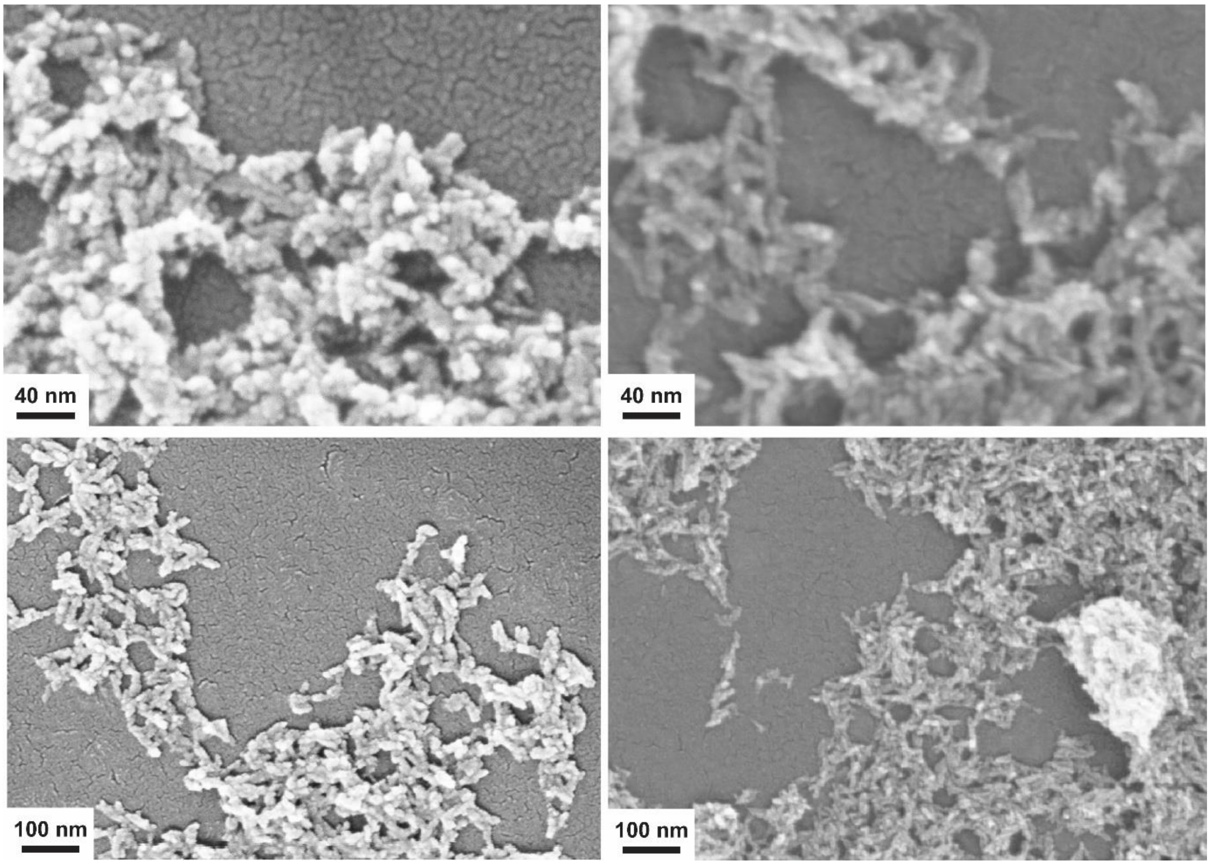

Figure S9: SEM images of L*-threoninol-functionalized* TiO_2 *nanoparticles after synthesis (left) and nanoparticles recovered after grinding the aerogel (right).*

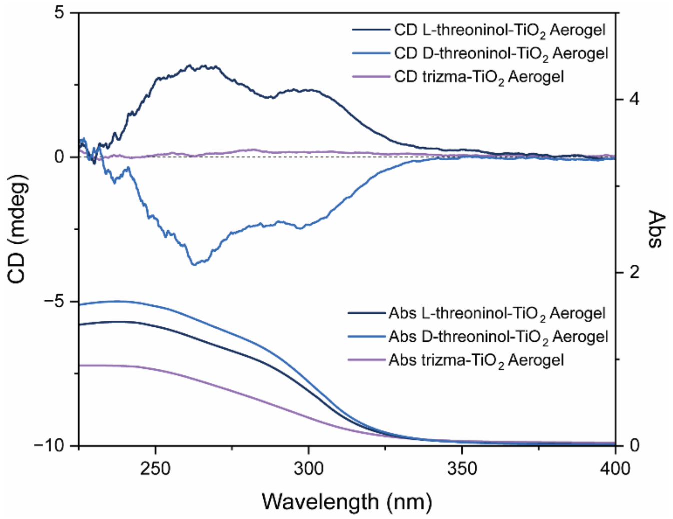

Figure S10: CD spectra and UV–vis spectra of L*- and* D*-threoninol-functionalized* TiO_2 *aerogels and trizma-functionalized* TiO_2 *aerogel.*
